# A Quality Improvement Project on Team-Based Care for Depression Screening Before and During the COVID-19 Pandemic in a Specialty Clinic

**DOI:** 10.7759/cureus.74234

**Published:** 2024-11-22

**Authors:** Alvina D Brueggemann, Peter G Harper, Holly Boyer, Shannon Fjestad, Lynn A Burmeister

**Affiliations:** 1 Quality Improvement, M Health Fairview, Minneapolis, USA; 2 Family Medicine, University of Minnesota School of Medicine, Minneapolis, USA; 3 Otolaryngology, University of Minnesota School of Medicine, Minneapolis, USA; 4 Clinical Informatics, M Health Fairview, Minneapolis, USA; 5 Endocrinology, Diabetes and Metabolism, University of Minnesota School of Medicine, Minneapolis, USA

**Keywords:** covid-19 impact of lockdown, depression in diabetes, depression screening, quality improvement projects, specialty healthcare

## Abstract

Background: Depression screening is an important first step to identifying patients who might benefit from depression treatment. Merit-based incentive payment system (MIPS) quality measures can yield financial benefits or losses for healthcare systems, including depression screening.

Objectives: This study aims to (1) develop a team-based care workflow to improve MIPS depression screening in a specialty clinic and (2) modify the workflow to include a virtual nursing and behavioral health resource after the COVID-19 pandemic hit.

Methods: A quality improvement project, utilizing Lean Six Sigma process improvement methods, was implemented to improve team-based depression screening in a specialty clinic. A multidisciplinary team implemented plan-do-study-act cycles, created educational materials tailored to each role, developed electronic medical record (EMR) tools to alert and assist team members in screening, and ensured the EMR aligned with the MIPS criteria. The percentage of eligible visits where depression screening was performed was analyzed across four study periods: pre-intervention, post-intervention, COVID-19, and recovery. Recovery strategies included developing telehealth workflows, establishing centralized registered nurse triage groups, and using phone-based triage and support resources at virtual visits.

Results: Utilizing team-based strategy and available or newly developed tools, the percentage of eligible visits with completed depression screening was as follows: 1.4% pre-intervention, 58.2% post-intervention, 3.5% COVID-19, and 64.0% recovery. With the COVID-19 pandemic outbreak, initially, improved screening performance declined sharply. Recovery was achieved through the revision of workflows, team members, and support tools.

Conclusions: A team-based care approach can successfully improve and maintain depression screening in a specialty clinic and was versatile enough to be readapted to virtual visits during the COVID-19 pandemic. In addition to impacting MIPS quality incentives, the depression screening workflows described in this article can be adapted to other uses, including virtual and in-person visits and in other specialty or chronic disease settings.

## Introduction

Depression affected 19.4 million adults in the United States [1] and 280 million people worldwide in 2019 [2]. Depression can have a significant emotional and physical impact on a person’s life, as well as financial cost. The economic burden of major depressive disorder was an estimated $236 billion in 2018, including direct medical costs, workplace costs (such as absenteeism and presenteeism), and suicide-related costs [3].

Depression can affect patients’ adherence to medication regimens, disease management, diabetes-related complications, risk of mortality, quality of life, and health care costs [4,5]. Patients struggling with depression have a 60% increased risk of developing type 2 diabetes [6] and an increased risk of cardiovascular disease [7].

It is estimated that 50-73% of patients (adults and children) go unrecognized or inadequately treated for serious mood disorders, including depression [8]. Screening for depression in the adult population is recommended by the US Preventive Services Task Force [9], the Institute for Clinical Systems Improvement [10], the American Diabetes Association [11], the American Association of Clinical Endocrinologists [12], and other professional societies.

The merit-based incentive payment system (MIPS) adjusts Medicare Part B payments based on performance in four performance categories: quality, cost, promoting interoperability, and improvement activities. Depression screening is one of the MIPS quality measures incentivizing healthcare systems to perform preventive depression screening. Despite the importance of attaining quality metrics, meeting them can be challenging for institutions. Innovative approaches are needed to improve MIPS quality scores and financial enhancements.

While primary care providers often conduct depression screening [13-15], it is important to recognize and utilize any and all touchpoints across the system, including when patients do not have or regularly visit a primary care provider. Depression screening can be conducted in specialty settings as well, giving providers more opportunities to identify patients who need additional support [16-19].

In January 2019, a specialty clinic was performing below the 20th percentile on MIPS depression screening and follow-up. A formal process was rolled out to improve depression screening. The first half of this paper focuses on this improvement in MIPS depression screening.

However, in 2020, the same clinic encountered a setback. A worldwide pandemic emerged, COVID-19, forcing clinics and healthcare systems to transition to virtual settings [20]. The completion of the depression screening process plummeted during this time as providers had to determine entirely new processes to deliver care to patients virtually. The second half of this paper describes a process that was developed to ensure virtual resources were available to patients who scored high on depression screening and needed additional support.

This study aimed to (1) develop a team-based care workflow to improve MIPS depression screening in a specialty clinic and (2) modify the workflow to include a virtual nursing and behavioral health resource after the COVID-19 pandemic hit. The depression screening workflows described in this article are versatile and can be adopted in many different team-based settings, including virtual and in-person visits.

## Materials and methods

Ethical considerations

The study was reviewed by the University of Minnesota Institutional Review Board and determined not to be human research (approval number: STUDY00016933).

Context

This project was implemented in an endocrine specialty clinic. The clinic is set within a multi-specialty ambulatory building and sees over 9000 patients annually. The clinic team includes physicians, advanced practice professionals, registered nurses (RNs), and medical assistants (MA). The system uses the Epic electronic medical record (EMR).

While standardized depression screening workflows had been implemented years prior, performance continued to be low. Therefore, the specialty clinic participated in the Impact Program, a five-month program designed to increase performance improvement capability at the practice level by using Lean methodology and the lens of team-based care. The Impact Program was sponsored by the Transforming Clinical Practice Initiative, implemented by Vizient, and funded by the Centers for Medicare and Medicaid Services.

Interventions

A multidisciplinary team from the specialty clinic, including physicians, nurses, MAs, administrators, Epic builders, informatics, and quality improvement consultants, was formed to develop, implement, and monitor the intervention. Using Lean methodology, the team reviewed the current process, designed and vetted the future process based on the MIPS depression screening criteria, and developed interventions. The team implemented plan-do-study-act (PDSA) cycles, created educational materials tailored to each role, developed EMR tools to alert and assist team members in screening, and ensured that the EMR was aligned with the MIPS criteria.

In the new process, depression screening was implemented through a team-based care approach. Epic Health Maintenance Alert (HMA) alerted staff to perform depression screening on eligible patient visits if it had not been done within the calendar year. The screening was performed by the MA during the rooming process. First, the two-question Patient Health Questionnaire-2 (PHQ2) was verbally administered. If the PHQ2 was positive (score > 3), then seven additional questions were asked to complete the Patient Health Questionnaire-9 (PHQ9). If the PHQ9 was positive (total score > 10 or a positive answer in Question 9), the clinic RN or provider would further assess the depression status using a smart-text phrase to guide suicide screening using the Columbia Suicide Severity Rating Scale. Clinical decision support was built into the smart text, which outlined various recommended follow-up options ranging from simple crisis resources printed in the after-visit summary to contacting the primary care physician, presenting the case to a behavioral health clinician, referring to an existing mental health provider, or considering sending the patient to the emergency room. RN completed suicide screening results were discussed with the provider, and a plan was developed and approved by the provider. The provider would review the plan with the patient, discuss basic safety plans, and document all screenings and follow-up plans in the EMR.

Significant modifications were made to align the Epic HMA display with the MIPS depression criteria to determine which patient visits were due for screening. Modifications included programming the Epic HMA to update every calendar year (rather than individual rolling 12-month dates), ensuring correct visit types and providers were included, having the proper patient exclusion criteria, and developing a way to document patient refusals to screen. By the second PDSA cycle, notification of eligible patients was moved away from the HMA, which required extra clicks and additional navigation, to a tab directly in the staff’s regular rooming process for easy viewing and access (rooming-based navigator). The tab appeared if the patient was due for screening and did not appear if the patient was not eligible for depression screening.

Initial pilots and training

Pilot versions of the new process occurred over two months in March-April 2019 with many changes made to Epic and its functionality. Personnel training was done rapidly over two weeks in March 2019 with an all-staff meeting to review the workflow in Epic, follow-up steps, and available behavioral health resources. In addition, there was a short video about the Columbia Suicide Severity Rating Scale for nurses and providers, along with discussions with behavioral health clinicians about dealing with depressed patients and asking sensitive questions. One-on-one hands-on training was provided, and staff underwent process reviews with clinic supervisors for the first several patients. Tip sheets, badge buddies (reminders attached to staff identification badges), and contact information for behavioral health providers were all made accessible to all staff and clinicians.

Improvement process

Clinic staff discussed progress and reviewed data daily at a huddle board. The clinic manager and quality improvement consultants worked with team members to identify and solve problems. The clinic manager and executives would meet every other week to review progress and identify resources for unsolved problems. Health system executives would also round with frontline staff monthly to problem solve, strengthen communication, and increase the visibility and importance of the work.

COVID-19 impact

With the beginning of the COVID-19 pandemic in March 2020, in-person clinic visits stopped and were immediately replaced by virtual visits. This required a quick pivot requiring the design, testing, and implementation of new processes to perform depression screening in the virtual setting. MAs could still verbally do the PHQ2 and PHQ9 in a video or phone visit. Positive PHQ9 screens were now escalated to a centralized virtual RN triage group instead of the clinic RN. The triage group used a more in-depth assessment tool (starting with the Briggs Telephone Triage Protocols for Nurses in 2019, then transitioning to the Schmitt-Thompson protocol in 2020) to assess risk and provide treatment options, including virtual behavioral health referrals. In addition, trained triage nurses updated the patient’s appointment note with information about the disposition of the patient (e.g., was the patient okay to continue with a specialty visit or did the patient need to seek emergency treatment).

Measures/data collection

The intervention was monitored using a process measure that was collected, graphed as a run chart, and posted to the clinic team with daily feedback discussing how to solve problems and improve the process. The depression screening compliance rate was calculated as the percent of eligible visits successfully screened with the PHQ2 and the PHQ9 (if the PHQ2 was positive). Data for the depression screening compliance rate was obtained from weekly Epic reports documenting whether the PHQ2/PHQ9 was performed on each eligible patient visit.

Analysis

Screening compliance rate was plotted on a control chart over time in four phases: pre-intervention, post-intervention, COVID-19, and recovery. Time frames for each phase were as follows: pre-intervention spanned January 2018 to February 2019 (14 months), post-intervention covered March 2019 to March 2020 (13 months), the COVID-19 phase lasted from April 2020 to October 2020 (7 months), and the recovery phase extended from November 2020 to April 2022 (18 months).

Process change over time was graphically depicted on a p-chart, which graphed the proportion of eligible visits correctly receiving depression screening. A Laney-P-chart was used since the measure exhibited overdispersion. Proportion tests (two-proportion tests) were used to compare depression screening rates across phases. A p of < 0.05 is considered significant.

## Results

**Figure 1:**
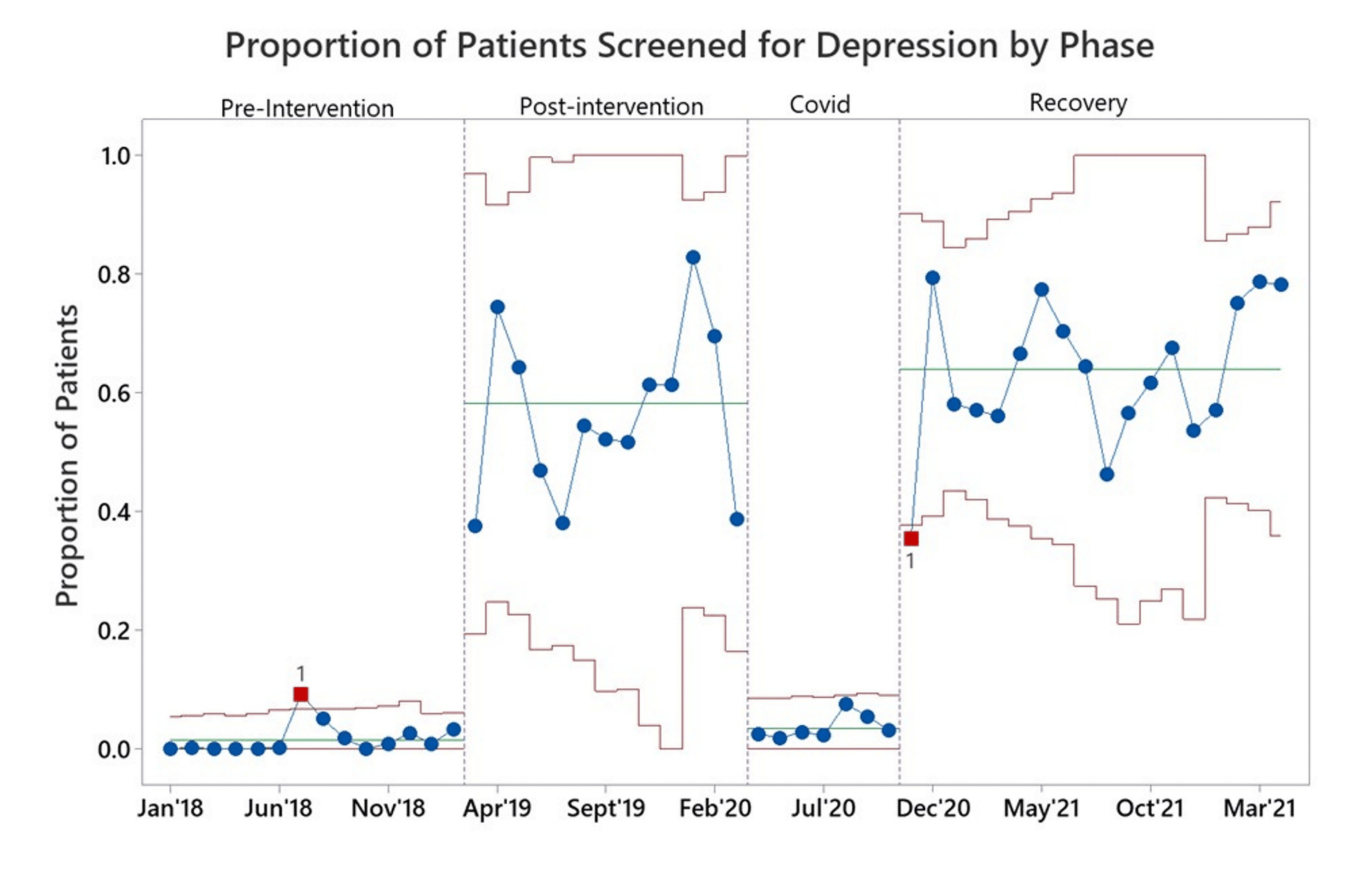
Control chart: depression screening compliance Laney-P-chart showing the proportion of eligible visits for which depression screening was completed over the four time phases of the study. Blue lines indicate the percent screened at each time point, green lines indicate the mean percent screened during the study interval, and red lines indicate the 95% confidence intervals.

During the entire 52-month study period, 19485 visits were eligible for depression screening, including 6951 during the 14-month pre-intervention period, 5423 during the 13-month post-intervention, 2450 during the seven-month COVID-19 impact period, and 4661 during the 18-month recovery period. The percentage of eligible visits for which depression screening was completed during each of the four phases of the study is shown in the control chart in Figure 1.

The average percentage of eligible visits for which depression screening was completed during each phase was 1.4%, 58.2%, 3.5%, and 64.0%, respectively (Table 1).

**Table 1:** Eligible percentage screened for depression n: number

Phase	Eligible for screening (n)	Screened (n)	Percent screened
Pre-intervention	6951	100	1.4%
Post-intervention	5423	3154	58.2%
COVID-19	2450	86	3.5%
Recovery	4661	2982	64.0%

Significant differences in the percentage of visits for which depression screening was completed were noted across the study phases. A two-proportion test indicated that the pre-intervention rate of screening (1.4%) was significantly lower than the post-intervention rate of screening (58.2%, p<0.001). The rate of depression screening then dropped significantly during COVID-19 (3.5%) compared to post-intervention (p<.001). A final two-proportion test indicated that depression screening rates rose significantly post-COVID-19 to 64% in the recovery phase (p<0.001).

## Discussion

This study shows that a team-based care approach can successfully improve and maintain depression screening rates in an endocrine specialty clinic. There was a statistically significant increase in depression screening from 1.4% to 58.2% (p<0.001) after the intervention was implemented. The COVID-19 pandemic resulted in a sharp decline in depression screening performance. After the difficult task of pivoting to virtual visits, new processes once again resulted in a statistically significant improvement in depression screening from 3.6% to 64% (p<0.001).

Team-based care to address poor adherence rates to clinic processes has successfully improved medication reconciliation, preventive care screening, and chronic pain care. Inter-professional teamwork can help providers meet the challenges of the Quadruple Aim [21].

Depression screening does not have to be a solo endeavor. As we did in this study, depression screening may be team-based, using MAs as the foundation to perform the initial tasks and then escalating care to other team members (RN, provider) as needed. This results in the efficient use of team members, improved team functioning, and improved care for patients. In the past, MAs only performed basic rooming and provision of services after receiving physicians’ orders. However, the increased role of the MA in the Share the Care model, as conceived by Bodenheimer et al. [22], has been effective in addressing primary care tasks including visit assistance [23], preventive services [24,25], and visit wrap-up [21]. MA-driven protocols have been successful in achieving greater colonoscopy orders [25] and mammogram rates [24], in addition to other preventative health initiatives.

Important elements of successful team-based care (as described in the “Share the Care” model) [26] include mapping team workflows, training, regular team meetings, ground rules, colocation, concrete goals agreed on by all team members, and standing orders. Team-based care can be effective in specialty as well as primary care settings and has been shown to be associated with reduced blood pressure, lipids, and glucose in cardiovascular and diabetes patients [27,28].

Depression screening has been performed successfully in a team-based fashion at specialty medical clinics. Bruschwein et al. [16] developed a process where outreach screening for depression was conducted by a doctoral-level psychologist or a social worker for every cystic fibrosis patient. Peters et al. [19] implemented a team-based approach where depression screening was added to a transplant nurse coordinator checklist used at every patient check-in. These approaches, while thorough and effective, may require extensive specialized resources, such as social workers and nurse coordinators.

In an oncology clinic, Gerard et al. [17] implemented a process where depression screening was completed either by patients on a welcome tablet prior to the visit or by a medical office assistant or RN at the beginning of the visit. Patients with significant positive responses on depression screening were able to discuss treatment and/or referral options with a provider. This process is a useful model for specialty clinics that do not necessarily have a dedicated behavioral health or care coordinator/outreach specialist onsite. This type of workflow was developed and used as a model at the endocrine clinic.

This study also showed the dramatic impact of the COVID-19 pandemic on clinic processes. The new process had significantly improved depression screening rates, but the early COVID-19-19 pandemic erased these gains. Using Lean Six Sigma methodology, the team was able to revamp the depression screening process to accommodate a virtual visit. This work once again improved depression screening even beyond previous levels. One of the major innovations implemented in virtual screening was the use of a virtual RN phone pool for patients who screened positive for depression.

One of the more difficult issues encountered in the implementation of the process was the concerns of RNs and specialty providers handling depression issues. Changing depression “assessments” to “screenings” improved RN acceptance. Providing education, training, and easy connections to behavioral health clinicians helped build comfort and skills for RNs and providers. Medical specialty providers were reassured that they were not expected to treat depression; rather, they were encouraged to adhere to evidence-based protocols to recommend next steps, provide resources, and/or place mental health referrals to meet patient care needs.

A critical challenge was making the EMR identify eligible patient visits and providing decision support and resources at the appropriate junctures. Significant work was done to define an eligible patient visit by calendar year versus a rolling 12-month alert. This was needed to meet the MIPS criteria. Work was also done to provide the alerts at the proper time and location on the screen and to embed the screening into the rooming staff’s regular rooming process. Having an EMR expert on the QI group was crucial to building a workable system.

Limitations

This study does not address the clinical outcomes of depression screening. This project only measures whether the process of depression screening could be successfully implemented in a specialty clinic. Other studies are needed to determine the effectiveness of screening on health outcomes. However, a reliable process for depression screening is the first step in the treatment of depression, which has been shown to enable patients to achieve optimal health.

Another limitation of this study is assessing the follow-up of positive PHQ9 screens. We did monitor the number of positive screens and the follow-up. However, the data were small enough that statistical analysis was not justified.

## Conclusions

This study shows that a team-based care approach can successfully improve and maintain depression screening in a specialty clinic and was versatile enough to be readapted to virtual visits during the COVID-19 pandemic. Hopefully, this work will contribute to the identification and treatment of depression in patients with chronic diseases, in addition to impacting MIPS quality incentives. Further work is needed to spread this work to other specialty clinics.

Team-based care is an effective way to accomplish population-based health endeavors in the clinical setting. Processes can be adapted in times of need. For instance, in the transition to virtual settings, team-based care can extend beyond in-person resources to include virtual resources such as centralized call centers or triage nurses. Depression screening in specialty clinics is possible, and improvements in depression screening and other ambulatory screenings may lead to both improved outcomes for patients and increased MIPS reimbursement for healthcare systems.
